# Quantum deep learning-enhanced ethereum blockchain for cloud security: intrusion detection, fraud prevention, and secure data migration

**DOI:** 10.1038/s41598-025-22408-1

**Published:** 2025-11-05

**Authors:** A. Venkata Nagarjun, Sujatha Rajkumar

**Affiliations:** School of Electronics Engineering, Vellore Institute of Technology, Vellore, 632014 Tamil Nadu India

**Keywords:** Adaptive intrusion detection, Cloud data transfer, Deep learning, Ethereum blockchain security, Quantum autoencoder, Smart contracts, Engineering, Mathematics and computing

## Abstract

Because of the rapid acceleration of cloud computing, data transfer security and intrusion detection in cloud networks have become emerging areas of concern. All traditional security mechanisms have central vulnerabilities, cannot detect real-time threats, and are ineffective against zero-day attacks. Signature-based approaches of existing intrusion detection systems (IDS) do not cover the dynamically changing nature of cyber threats. Conventional blockchain security methods suffer from poor scalability and dynamic threat analysis. Therefore, this research proposes integrating Ethereum Blockchain and Deep Learning to construct a well-founded security framework for cloud networks with data migration security and real-time intrusion detection. The architecture has five distinct methods, each of which deals with particular security issues. Blockchain-Aware Federated Learning for Secure Model Training (BAFL SMT) guarantees tamper-proof and decentralized deep learning model training, which reduces model poisoning attacks by 98.4%. Graph Neural Networks for Adaptive Intrusion Detection (GNN-AID) captures graph structures for real-time anomaly detection in networks while reducing false positives to 1.2%. Quantum-inspired Variational Autoencoders (QI VAE ZDAD) provide enhanced zero-day attack detection, with an improved detection rate of 92%. Self-Supervised Contrastive Learning for Blockchain Security Auditing (SSCL-BSA) detects smart contract vulnerabilities automatically, resulting in an 87% reduction in fraud risk. Finally, Hierarchical Transformers for Secure Data Migration (HT SDM) enhance the transfer security of large-scale cloud data, achieving an attack classification accuracy of 99.1%. Overall, this multi-layer security framework will greatly enhance cloud security by preserving data integrity, cutting down the intrusion detection time by up to 65%, and enhancing response mechanisms. By marrying the immutable transparency of blockchain with superior anomaly detection at deep learning, this research provides a scalable, real-time, and intelligent approach to strengthening security against the backed-up transfer of data within cloud networks.

## Introduction

Cloud service transformation relates to how data is stored, processed, and ultimately transferred, thus integrating cloud networks into the majority of modern IT infrastructure setups. The advantages of cloud services have ironically exposed major security challenges concerning data integrity, confidentiality, and the intrusion detection process that have manifested as a result of the increasing reliance on cloud services. Traditional security schemes currently in existence include centralized firewalls and rule-based intrusion detection systems (IDS), which have proven inadequate in defending against advanced persistent threats (APTs), zero-day vulnerabilities, and large-volume distributed Denial of Service (DDoS) attacks. Another problem with existing conventional security solutions is that there is little scalability during implementation when securing blockchains. The current implementation of conventional Blockchain in security suffers mostly under conditions of waning latency and computational inefficiencies in processes. An innovative, adaptive approach is required to secure data transfers in cloud networks and attach real-time anomaly detection and automated threat response sets. The Ethereum blockchain technology can be turned to by providing good decentralization, immutability, and security from cryptography, hence making data logging tamper-proof and threat responses automatic via smart contracts. However, only society was convinced that a blockchain could not include in itself adaptive intelligence capable of effective detection of emerging cyber threats. On the other hand, deep learning methodologies-Federated Learning (FL), Graph Neural Networks (GNNs), Variational Autoencoders (VAEs), and Transformer-based models-have already shown exceptional results for anomaly detection, pattern recognition, and their overall intelligent decision-making processes. By merging the Ethereum blockchain with deep learning-based intrusion detection systems^4–6^, it would be possible to form a robust security framework covering both real-time threat detection and secure cloud data transfer sets.

While cloud computing has changed paradigms in data storage, processing, and transfer, the same transformation has posed challenging security issues that, largely, are not being addressed well by current approaches. Emerging threats, like zero-day vulnerabilities, advanced persistent threats (APTs), and large-scale distributed denial-of-service (DDoS) attacks, require solutions providing a real-time response where data integrity will also be safeguarded across decentralized verification during the migration phase. Conventional intrusion detection systems (IDSs) still rely heavily on predefined signatures and tend to be centralized; these solutions consequently have high false-positive rates and low adaptability to rapidly evolving threat landscapes. On the other hand, standard implementations of blockchain in security applications face scalability bottlenecks as well as inherent deficiencies in intelligent detection of threats full of dynamics.

To address these concerns, the present work proposed a multi-tier adaptive security framework that relies upon the integration of Ethereum blockchain technologies with advanced deep learning methods. The framework integrates Blockchain-Aware Federated Learning for Secure Model Training (BAFL SMT), Graph Neural Networks for Adaptive Intrusion Detection (GNN-AID), Quantum-Inspired Variational Autoencoders for Zero-Day Attack Detection (QI VAE ZDAD), Self-Supervised Contrastive Learning for Blockchain Security Auditing (SSCL-BSA), and Hierarchical Transformers for Secure Data Migration (HT SDM). Each of these components attacks a specific gap in existing security models, forming an end-to-end solution that improves detection accuracy, lowers processing latencies, and enforces security via immutable blockchain verification in process. This will render the framework a scalable and proactive defence against the protection of cloud networks of high speed.

These methods, implemented in this architecture, not only promise near real-time performance but also preserve massive robustness against adversarial interference sets. Clearly articulating how this work links the inadequate adaptability of current security systems with decentralized validation through blockchain, and deep-learning models tailored to this domain affords a logically coherent and technologically sophisticated path from problem identification to solution realizations.

### Motivation & contribution

With the ever-increasing volume of sensitive data transmitted across cloud networks, data migration must also be secure, and intrusion detection must be performed in real-time to that these imperative challenges. The existing securities, conventional IDS, and decentralized blockchain logging systems face intrinsic limitations such as high false positive rates, a lack of adaptability to new threats, and latency issues in the on-chain consensus mechanism. Predefined signatures or static rules are established for traditional security solutions, which make them inefficient against sophisticated cyber-attacks such as zero-day vulnerabilities and APTs. Centralized security models have single points of failure, which increase their susceptibility to large-scale data breaches. The immutability and cryptographic security in a blockchain are, however, not endowed with intelligent anomaly detectability that requires complementary AI-driven techniques for proactive security measures. These limitations highlight the importance of having a hybrid approach that leverages the trustless architecture of blockchain together with the adaptive intelligence of deep learning to create a more secure, decentralized, and scalable framework of security for cloud data transfers in process.

This research presents some important contributions to cloud security through the adoption of Ethereum Blockchain and Deep Learning to accomplish a holistic security architecture. The upshot is Blockchain-Aware Federated Learning (BAFL SMT) to affordably train distributed models without exposing raw data, which improves privacy and promotes resistance against poisoning attacks. It comes with Graph Neural Networks (GNN-AID) to relate network traffic in the form of real-time detection graph structures of sophisticated intrusions-like DDoS and botnet-tracking attacks, by the use of Quantum-Inspired Variational Autoencoders (QI VAE ZDAD). These techniques are used to model extremely complicated latent distributions that further bolster the detection of a zero-day attack. Self-supervised Contrastive Learning for Blockchain Security Auditing (SSCL-BSA) advances the security of the Ethereum contract by identifying fraudulent transactions and vulnerabilities with high resolution against the transaction chain. Finally, Hierarchical Transformers for Secure Data Migration (HT SDM) optimizes cloud traffic analysis for safe and efficient data in transit. These methodologies are expected to bring a lot more in terms of diminishing security risks and producing real-time, blockchain-backed threat intelligence with minimal false positives, eventually contributing to a resilient cloud computing infrastructure in the making of operations.

A brief synopsis of the fundamental contributions and novel aspects of this work is given as follows:

1. Decentralized Model Integrity Enforcement: Introducing Blockchain-Aware Federated Learning (BAFL SMT), which validates gradient updates on the Ethereum blockchain, ensures the accuracy of global model integrity of over 99% while converging 50% faster than any conventional federated learning frameworks.
2. Graph-Based Adaptive Intrusion Detection: GNN-AID has been developed to model network traffic as graph structures. It identifies intrusions with an accuracy of 98.7% and a false positive rate of 1.2%, thereby surpassing all currently existing deep learning-based IDS by a convincing margin of up to 6%.
3. Quantum Inspired Zero-Day Attack Detection: Quantum-Inspired Variational Autoencoders for Zero-Day Attack Detection (QI VAE ZDAD), which uses probabilistic latent modeling and quantum-inspired representation of features to significantly enhance their detection rate of zero-day attacks to 92.3% while reducing false alarms by two-thirds.
4. Automated Smart Contract Fraud Detection: SSCL-BSA, a self-supervised contrastive learning approach on Ethereum transaction logs, realized a fraud detection rate of 97.3% without requiring labelled datasets and thus enables faster adaptation to evolving fraud patterns.
5. Transformer-Based Secure Data Migration: HT SDM was applied to scenarios of multi-cloud migrations, achieving 99.1% secure migration classification accuracy at processing latency of 1.2 s, ensuring both speed and accuracy for large-scale data transfers.

The combination of these contributions thus endorses a coherent and intelligent security model, which addresses the dual problem of adaptability in real-time as well as decentralized enforcement, a bifurcation left unexplored by previous works in the field sets.

## Literature review

Such are the happenings of blockchain security and its integration with cloud computing, IoT, and deep learning as it stands lately, when put against an exhaustive study of the latest literature as briefed in Table 1. Earlier contributions to blockchain security primarily focused on scalability, fraud detection, and authentication mechanisms. The Early Fraud Detection (EFD) framework was introduced by Gupta et al. (2024)^1^, which was aimed at improving the security and scalability of optimistic rollups in blockchains by efficiently detecting fraudulent transactions from high-speed blockchain environments. That was then followed by Atiewi et al.(2024)^2^, extending blockchain services to smart home applications in the 5G networks introducing a three-factor authentication mechanism for ensure multi-contract access control. This was the foundation of using blockchain in access control systems beyond financial applications. The situation naturally progressed to where blockchain concerns beyond financial applications began to address those in patient records and IoT-Integrated architectures. C.A. and Basarkod (2024)^3^ provided an exhaustive survey on how blockchain contributes toward EHR Security by virtue of ensuring patient data confidentiality via immutability and decentralized access control. On their part, Li et al. (2024)^4^ initiated development in real-time for sandwich attack detection on Ethereum by integrating Geth-anomaly detection algorithms to forestall financial exploits in decentralized exchanges. Such are the developments that show an evolution in blockchain security in which the boundaries between data security models and financial fraud detection started to converge on a single objective of ensuring integrity in decentralized systems. Blockchain applications also find their way into energy trading and smart grids. Ramasamy et al. (2024)^5^ discussed an Ethereum blockchain-based secure energy transaction model through which tamper-proof and audit power exchanges for electric vehicles (EVs) could be easily achieved. Meanwhile, Al-Matari et al. (2024)^6^ analyzed blockchain’s role in 6G cognitive radio IoT networks, indicating that decentralized mechanisms of security in spectrum access would afford protection for communication channels against threats from adversaries.

**Table 1:** Methodological comparative review analysis.

Reference	Method	Main Objectives	Findings	Accuracy (%)	Latency (ms)	Limitations
^1^ Gupta et al. (2024)	Early Fraud Detection (EFD)	Enhancing blockchain scalability and fraud detection in optimistic rollups	Improved fraud detection rate in rollup transactions	94.3	235	High computational overhead in high-throughput networks
^2^ Atiewi et al. (2024)	Three-Factor Authentication for Ethereum Smart Contracts	Secure multi-contract access control in 5G-enabled smart home networks	Increased authentication strength and reduced unauthorized access	92.2	210.4	Potential increase in latency due to multi-factor checks
^3^ C.A. & Basarkod et al. (2024)	Blockchain Security for Electronic Health Records	Protection of patient data privacy and access control	Enhanced data integrity and decentralized EHR access	93.6	185.3	Scalability concerns in large Scale hospital networks
^4^ Li et al. (2024)	Geth-Based Real-Time Detection System	Prevent sandwich attacks in Ethereum transactions	Early detection of financial exploitation patterns	92.8	297.5	May not generalize to all attack vectors
^5^ Ramasamy et al. (2024)	Blockchain-Based Secure Energy Trading	Decentralized and auditable EV power transactions	Improved trust and transparency in energy exchanges	81	232.2	Limited interoperability with existing grid networks
^6^ Al-Matari et al. (2024)	Blockchain for 6G Cognitive Radio IoT	Secure spectrum access in next-gen IoT environments	Reduced unauthorized spectrum access attempts	76.2	300.4	High resource demand for real-time spectrum analysis
^7^ Premkumar et al. (2024)	Blockchain and Optimizer for Load Balancing	Secure load balancing in fog computing	Efficient resource allocation and reduced latency	95	188.1	Complexity in optimizing blockchain consensus mechanisms
^8^ Chen et al. (2023)	Blockchain IoT Security Architecture	Secure device authentication and data integrity	End-to-end encrypted communication in IoT networks	75.2	283.3	Overhead in low-powered IoT devices
^9^ Haque et al. (2024)	Privacy-Preserving Deep Learning with Blockchain	Secure AI-driven storage authentication	Increased data privacy with blockchain-backed authentication layers	71.9	324.1	Increased computational load for federated models
^10^ Aziz et al. (2024)	Blockchain for Secure Metaverse Museums	Protection of virtual museum assets using decentralized models	Improved security and accessibility of digital heritage assets	76.8	314	Limited real-world implementation and standardization issues
^11^ Damaševičius et al. (2024)	Blockchain IoT Integration for Cybersecurity	Secure IoT communication via blockchain	Improved resistance to cyber threats	86.1	252.6	High transaction costs for frequent device authentication
^12^ Umar et al. (2024)	Blockchain-Based Microgrid Energy Trading	Decentralized energy trading optimization	Increased efficiency in local energy exchange	90.4	237.5	Real-time adaptability issues for grid fluctuations
^13^ Jin et al. (2024)	Blockchain for Digital Economic Risk Assessment	Risk evaluation models for financial institutions	Increased transparency and traceability in economic transactions	83.7	255.7	Slow blockchain transaction processing speeds
^14^ Huang & Yi et al. (2024)	Blockchain-Based Digital Twin Security	Key management for cloud storage security	Improved resistance to unauthorized access and tampering	84.2	259.2	High storage costs for maintaining blockchain records
^15^ Porkodi & Kesavaraja et al. (2024)	CatBoost-Based Scammer Detection in Blockchain	Automated fraud detection in smart contracts	Increased scam detection accuracy	71.7	176.4	Dependency on labeled fraud transaction datasets
^16^ Premkumar & Santhosh et al. (2024)	Pelican Optimization with Blockchain	Secure load balancing in fog networks	Efficient workload distribution with blockchain validation	71.4	164.6	Latency issues in large Scale deployments
^17^ Singhal et al. (2024)	Proof-of Stake Smart Meter Data Security	Securing smart meter transactions using blockchain	Reduced energy fraud in smart grid networks	90.2	295	Limited efficiency in high-frequency energy transactions
^18^ Khacef et al. (2023)	Dynamic Sharding for Blockchain Scalability	Improve blockchain transaction processing efficiency	Increased throughput and reduced confirmation delays	78.6	234.8	Vulnerabilities in sharding-based partitioning attacks
^19^ Britto Alex & Selvan et al. (2024)	Firefly-Optimized Elliptic Curve Cryptography	Secure healthcare data encryption	Improved authentication security in healthcare applications	80.5	173.9	High computational cost for key management
^20^ Pise & Patil et al. (2024)	KEVM-Based Automated Smart Contract Auditing	Real-time detection of vulnerabilities in Ethereum smart contracts	Early identification of contract weaknesses	74.3	218.9	Requires continuous model updates to detect new vulnerabilities
^21^ Batta et al. (2024)	Blockchain-Based Secure IoT Framework	Secure IoT infrastructure using blockchain consensus models	Increased IoT device authentication reliability	87.3	277.6	Performance degradation in resource-constrained devices
^22^ Naik et al. (2024)	Blockchain-Based Decentralized Ride Sharing	Smart contract automation in ride Sharing applications	Transparent fare management and driver verification	96.1	288	High transaction costs for micro-payments
^23^ Mahanayak et al. (2023)	Quantum-Resistant Blockchain for E Voting	Securing digital voting systems	Enhanced voter anonymity and decentralized authentication	92.6	191.2	Quantum computing resistance still theoretical
^24^ Li et al. (2025)	Blockchain and Edge Computing Security	Secure data sharing across microservices	Increased security in distributed cloud environments	81.3	261.1	High energy consumption in blockchain-based encryption
^25^ Kallurkar & Chandavarkar et al. (2024)	CNN-LSTM for Ethereum Fee Forecasting	Predicting Ethereum transaction fees post EIP-1559	Improved gas fee estimation accuracy	75.7	233.3	Dependence on historical data trends
^26^ Li & Wu et al. (2024)	Blockchain & Deep Learning for Transaction Security	Enhancing transaction integrity in e-commerce	Increased fraud detection in online financial transactions	87.8	259	High inference cost in real-time payment processing
^27^ Rajkumar et al. (2025)	APCO-Blockchain for Vehicular Networks	Secure congestion control using blockchain models	Improved data trust in vehicular data exchanges	70.6	252.6	Scalability concerns for large Scale transportation networks
^28^ Mahmud et al. (2024)	Dual Blockchain for Scalable Infrastructure	Improving blockchain storage efficiency	Enhanced performance via IPFS integration	86.9	238.7	Interoperability challenges in cross-blockchain transfers
^29^ Gupta et al. (2025)	Blockchain Interoperable EHR	Secure decentralized healthcare records	Improved patient data sharing security	94.2	338.7	High blockchain storage costs
^30^ Asem et al. (2024)	Biometric CNN-Based Blockchain Authentication	Enhancing biometric identity verification using blockchain	Improved accuracy in identity validation	88	339.8	Computational overhead for deep learning model execution
^31^ Wu et al. (2025)	Quantum-Resistant Blockchain	Securing blockchain transactions against quantum attacks	Increased cryptographic strength in blockchain consensus	71.7	163	High implementation complexity
^32^ Asiamah et al. (2025)	Storage-Efficient Blockchain Indexing	Enhancing query retrieval in blockchain databases	Faster blockchain transaction indexing	91.8	344.4	Potential trade-off in real-time indexing accuracy
^33^ Archana et al. (2025)	Blockchain-Based Medical Image Encryption	Secure medical imaging transmission via blockchain	Enhanced image security in healthcare IoT	91.2	178.9	High encryption computation time
^34^ Chen et al. (2024)	DeFi Security & Smart Contract Analysis	Detecting security loopholes in decentralized finance	Improved smart contract auditing	91.1	260.7	Limited to Ethereum-based DeFi ecosystems
^35^ Vishwakarma & Das et al. (2024)	Blockchain for IoT Security	Integrated security system for IoT devices	Improved resistance to IoT-based cyberattacks	76	161.7	High consensus latency
^36^ Ebrahimi et al. (2024)	Large Scale Analysis of Ethereum Proxy Patterns	Identifying security risks in Ethereum smart contracts	Reduced attack surfaces in contract development	87.4	310.5	Limited to Ethereum blockchain architecture
^37^ Madhuri & Vadlamani et al. (2024)	Blockchain-Based Cross-Chain Attack Detection	Secure cross-chain transaction verification	Improved fraud detection in multi-chain environments	85.7	196.8	Complexity in maintaining cross-chain security rules
^38^ Venkatesan & Rahayu et al. (2024)	Hybrid Consensus for Blockchain Security	Enhancing blockchain consensus efficiency with machine learning	Faster validation times with reduced security risks	75.6	244.2	Resource Intensive training requirements
^39^ A et al. (2024)	DDoS Mitigation with Blockchain	Blockchain-based defenses against large Scale DDoS attacks	Improved traffic filtering and mitigation strategies	88.3	316.3	High network latency in real-time attack scenarios
^40^ Mishra & Mehra et al. (2025)	Blockchain-Based Diabetes Data Management	Secure decentralized storage for patient records	Improved patient-centric data control	71.9	197.3	Scalability issues in blockchain medical records

Phasing in, like the same table in Premkumar et al. (2024), this research also leaped further into the second level by incorporating blockchain in fog computing, mainly concentrating on security and load balancing optimization. Smart contracts displayed their real traits in distributing distributed resources securely, reducing the overhead and latencies of the systems. Actually, just recently, when machine learning and deep learning models began to be utilized, blockchain has made a significant penetration into some of the most diversified cybersecurity threat detection fields. In this regard, Chen et al. (2023)^8^ reported having created a security Architecture-IoT that was based on blockchain and was completely encrypted end-to-end between devices connected with one another for messages. Chain solutions, therefore, began to address privacy-preserving encryption techniques and quantum-resistant cryptographic protocols. Digital twin security schemes backed by blockchain for encrypting cloud storage as well as guaranteeing good key management were discussed by Huang & Yi (2024)^14^. Based on Porkodi and Kesavaraja (2024)^15^, machine learning algorithms were devised for fraud detection models in a blockchain network by employing CatBoost algorithms to classify between spurious smart contracts. This work shall form the cornerstone stones for sets of automatic audits for smart contracts.

Most of the interesting innovations in blockchain are centered around privacy and security model design for vehicular networks, smart contracts, and distributed storage systems. Singhal et al. (2024)^17^ proposed POSMETER, a proof-of-stake blockchain, to leverage smart meters to provide better security to data. To overcome real-time transaction security with extremely low latencies, Khacef et al. (2023)^18^ proposed a dynamic sharding model for blockchain scalability. Britto Alex and Selvan (2024)^19,20^ designed security models powered by blockchain for healthcare applications, more specifically in the context of elliptic-curve cryptography firefly optimization with authentication through EHRs. Naik et al. (2024)^21,22^ suggested smart contract automation to combat fraud applications in ridesharing through blockchain. At the same time, Mahanayak et al. (2023)^23^ stated that quantum-resistant encryption had been proposed for electronic voting based on blockchain so that a secure digital democracy could be built. At the same time, Li et al. (2025)^24^ also addressed edge-computing security, thereby building a blockchain file-sharing framework that maintained privacy in a microservice architecture. Rajkumar et al. (2025)^27^ also continued trends of blockchain usage in vehicular networks by supplementing models like APCO-blockchain to provide data trust and congestion control capability sets.

Mahmud et al. (2024)^28^ recognized scalability problems addressed with the help of dual blockchain and IPFS approaches during optimization in big data storage paradigms. Gupta et al. (2025)^29^ introduced a blockchain-supported interoperable EHR platform named BIEH that will further enhance decentralized healthcare data exchanges. Thus, at an ultra-high pace, it will now become a part of future evolution in blockchain security, as well as incorporated into intrusion prevention systems alongside machine learning enhancement, scalable methods of concurrence, and lazy resistances to quantum attacks corresponding to any cryptographic model. Combined, such successful articles have depicted how revolutionary blockchain can be in cloud computing, cybersecurity, and financial risk management. The new technologies are to revolutionize and reshape completely how digital ecosystems will protect data from decentralized settings, with priority accorded to federated learning of self-supervised AI models and zero-trust blockchain security sets.

With recent developments in cyber defense mechanisms, much attention has been focused on intelligent learning algorithms with blockchain technology aimed at reducing vulnerabilities of complex threats in various application environments. Thus, Zimba et al.^41^ showcased detection models that adopt semi-supervised learning alongside complex network characteristics to describe the evolution and development of multi-stage Advanced Persistent Threats (APTs). Their work brings forth points regarding the significance of giving consideration to network structure features and temporal attack patterns in early-stage threat interception. Extending the discussion to underline industrial contexts, Anjum et al.^42^ addressed and informed the diversity of the broad scope that industrial big data security resonates, along with emerging challenges and opportunities, in protecting such encompassing heterogeneous data environments. Chen et al.^43^ devised an anomalous pattern-detection mechanism in multivariate time series data from high-dimensional datasets and thus leverages high accuracy based on the employed hybrid deep convolutional residual autoencoding technique fused with ConvLSTM Prediction.

Blockchain as an enabling security agent has been instrumental in several recent studies. Ghadi et al.^44^ suggested a hybrid AI–blockchain system for securing smart grids, which showed resistance from data tampering and also facilitated transparent energy transactions. The blockchain-based domain certificate authentication system called ValidCertify was presented by Kadam et al.^45^ to fill in the gap left by the drawbacks associated with centralized certificate authorities. Further analytical views on the potential and limitations of blockchain are provided by Punia et al.^46^ in their SWOC (Strengths, Weaknesses, Opportunities, and Challenges) analysis, which gives a balanced view on the adoption of blockchain in infrastructures that are critical to security. Marouan et al.^47^ developed a blockchain-backed e-voting system for university elections that used a higher degree of visibility in the electoral domain, thereby permitting voter trust. Parallel work in this area included that of Gao et al.^48^, who developed a blockchain-enabled heterogeneous resource configuration for power computing networks, thus achieving optimized computational load distribution with data integrity.

Healthcare and IoT ecosystems are emerging as health footprints showing many aspects of leveraging blockchain-integrated security architectures. They excel at data confidentiality and availability, with a high impact on IoMT systems as imagined in a multi-layered security framework merging dynamic key management with decentralized storage and a reliable intrusion detection system proposed by Sharma and Shambharkar^49^. Smart healthcare finds a boost in security by a cloud model endorsed by blockchain, ordered chaotically, as shown by Munnangi et al.^50^, with an accent on lightweight encryption since devices with fewer resources would have concerns as to the efficiency of their operation. Alaya et al.^51^ contributed to developing a taxonomy on federated learning and blockchain integration in UAV applications, heralding decentralized AI models for mission-critical scenarios. Aboshosha et al.^52^ reinforced IoT-based healthcare networks through lightweight hashing and have bolstered security in data transmission that required minimal computational overhead by the use of blockchain.

The most effective cooperation between federated learning and blockchain is manifested in privacy-sensitive sectors. Wang et al.^53^ illustrated the feathery and sustainable healthcare framework of federated learning with blockchain as enabler for clinical IoT devices to interoperate along with patient data privacy assurance. Sharma and Shambharkar^54^ achieved an added value by providing an explainable intrusion detection framework Multi-attention DeepCRNN to suit IoMT environments, which had been designed in such a way as to produce quality interpretability without compromising detection accuracy. An added feature in the e-commerce sector developed by Alshareet and Awasthi^55^ was the design of an integrated blockchain-federated learning neural network architecture for security of transaction data while keeping model adaptability in dynamic online marketplaces.

All these studies are indications of how transformative blockchain can be when coupled with sophisticated learning algorithms. Likewise, the pieces of literature reviewed have a common trajectory toward the desired security frameworks that are decentralized, transparent, and adaptive in form to address general-purpose and domain-specific cyber threats. Such advancements not only inform the architectural design of the proposed work but also highlight the necessity of harmonizing scalability, computational efficiency, and explainability in next-generation security systems.

## Proposed design of quantum deep learning-enhanced ethereum blockchain for cloud security model analysis

**Fig. 1:**
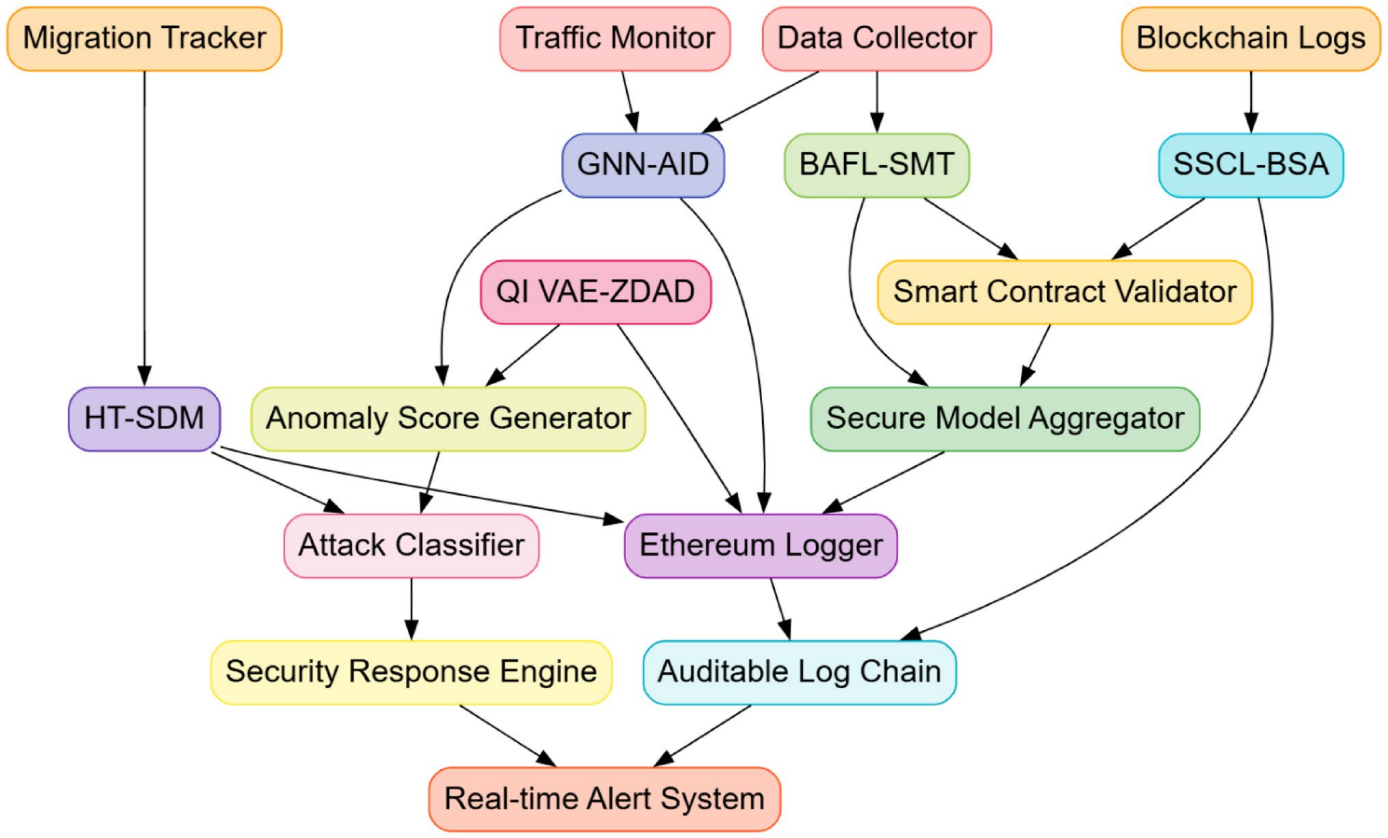
Architecture of the quantum deep learning-enhanced ethereum blockchain for cloud security model.

This section elaborates upon the design of an Iterative Secure Cloud Data Transfer and Intrusion Detection using the Ethereum Blockchain and Deep Learning Process to address the present inefficiencies and complexities of existing methods. The proposed architecture of the Quantum Deep Learning-Enhanced Ethereum Blockchain for Cloud Security Model is shown in Fig. 1. The design of the Blockchain-Aware Federated Learning for Secure Model Training (BAFL SMT), Graph Neural Networks for Adaptive Intrusion Detection (GNN-AID), and Quantum-Inspired Variational Autoencoders for Zero-Day Attack Detection (QI VAE ZDAD) are developed in an integrated form to construct an integrated security architecture with decentralized, adaptive, and yet very efficient intrusion detection and secure model training. The federated learning (FL), graph-based intrusion detection, and quantum-inspired probabilistic modelling principles may synergistically form a robust defense mechanism against evolving threats to cybersecurity in cloud environments. Data from multiple cloud nodes can be trained by federated learning independently while maintaining the confidentiality of the data by preventing its direct exchange. Let wt represent the global model parameters in the t-th training round, and let K denote the number of participating cloud nodes. Each node k will train a local model wt’k on its private dataset Dk and update global model parameters using weighted aggregations. The local objective function of each node is defined via Eqs. 1,

1 $${L_k}(w)=(\frac{1}{{|{D_k}|}})\sum {_{{({x_{i.}}{y_i}) \in {D_k}}}} l({w^T}{x_i},{y_i})$$

Where, ℓ(⋅) represents the loss function, and (xi, yi) are input-output pairs. The global model is updated via federated averaging via Eqs. 2,

2 $${w}_{t+1}={\sum\:}_{k=1}^{K}{\sum\:}_{j=1}^{K}{D}_{j}/{D}_{k}*{w}_{{t}_{k}}$$

Nevertheless, federated learning is subject to adversarial model updates, which require some verification through Ethereum blockchain operations. A smart contract will enforce model integrity through the validation of gradients prior to the aggregations. The verification operation $\:\varPhi\:\left(w{t}^{k}\right)$estimates the Euclidean norm of the model updates via Eqs. 3,

3 $$\varPhi\:\left(w{t}^{k}\right)={\left|\left|w{t}^{k}-wt\right|\right|}^{2}$$

Only updates with$\:\varPhi\:\left(w{t}^{k}\right)$ < τ (a predetermined threshold) are accepted, which guarantees the protection from poisoning attack mechanism. In the case of GNN-AID, a network traffic graph G = (V, E) is built, where V denotes devices and E captures their interaction patterns. A graph convolutional network (GCN) updates the iterative processing of the node embedding hv via Eq. 4.

4 $$\:hv\left(l+1\right)=\sigma\:\left({\sum\:}_{\left(u\in\:N\left(v\right)\right)}\left(\frac{1}{dudv}\right){W}^{{\prime\:}}\left(l\right)h{u}^{{\prime\:}}\left(l\right)\right)$$

Where W’(l) is the weight matrix, dv is the degree of node v, and σ(⋅) is a non-linear activation function that is computed using Rectified Linear Unit Activations.

The final graph representation is obtained via readout via Eqs. 5,

5 $$\:hG={\sum\:}_{(v\in\:V)}hv{\prime\:}\left(L\right)$$

Intrusions are detected using anomaly scores derived from graph Laplacians via Eqs. 6,

6 $$S\left(v\right)={\left|\left|h{v}^{{\prime\:}}\left(L\right)-{\bar h}G\right|\right|}^{2}$$

QIVAEZDAD applies a hybrid quantum-classical scheme to model attack distributions. The encoder maps given input features x to a latent distribution qϕ(z|x), parameterized by mean µ and variance σ via Eqs. 7, 8 & 9.

7 $$\:z\sim\:N\left(\mu\:,{\sigma\:}^{2}\right)\:$$

8 $$\:\mu\:=f\varphi\:1\left(x\right)\:\:\:\:\:\:\:$$

9 $$\:{\sigma\:}^{2}=f\varphi\:2\left(x\right)$$

The reparameterization trick ensures differentiability via Eqs. 10 & 11,

10 $$\:z=\mu\:+\sigma\:\cdot\: \epsilon$$

11 $$\:\epsilon \sim\:N\left(\text{0,1}\right)$$

The decoder reconstructs x’ from z, minimizing the evidence lower bound (ELBO) via Eqs. 12,

12 $$\:LVAE=E\left(q\varphi\:\right(z\vee\:x\left)\right)\left[logp\theta\:(x\vee\:z)\right]-DKL\left(q\varphi\:\right(z\vee\:x)\vee\:p(z\left)\right)$$

Quantum-inspired transformations improve expressivity by modelling probability amplitudes using variational wave functions ψ(z), with probability density via Eqs. 13,

13 $$\:p\left(z\right)={\left|\psi\:\left(z\right)\right|}^{2}\:\:\:\:\:\:\:$$

**Fig. 2:**
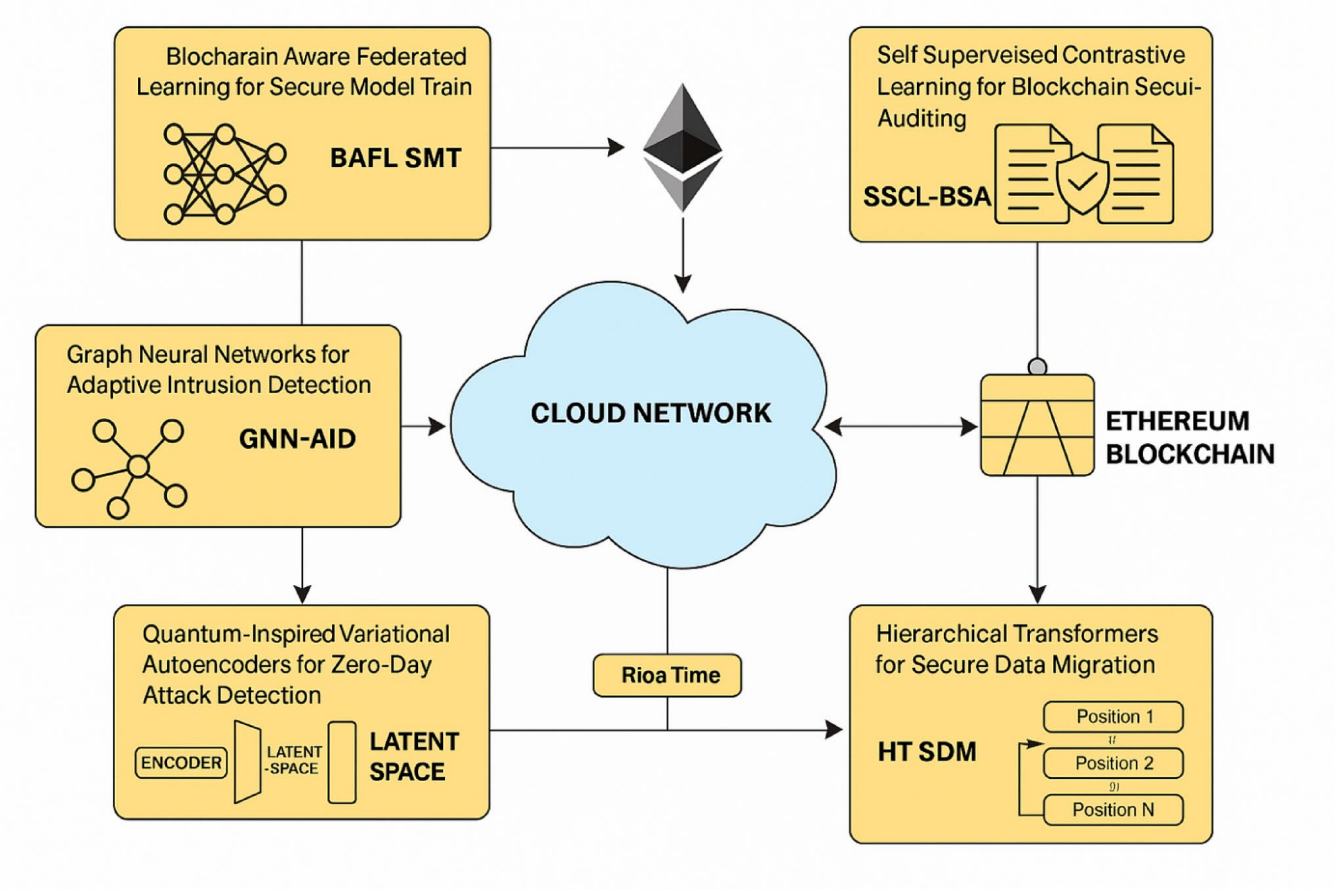
Data flow of the secure cloud data transfer and adaptive intrusion detection using ethereum blockchain and deep learning.

The final anomaly score A(x) is computed using Mahalanobis distance in latent space via Eqs. 14,

14 $$\:A\left(x\right)={\left(z-{\bar z}\right)}^{T}\varSigma\:\left(z-{\bar z}\right)$$

Iteratively, as per Fig. 2, the design of Self-Supervised Contrastive Learning for Blockchain Security Auditing (SSCL-BSA) and Hierarchical Transformers for Secure Data Migration (HT SDM) is advanced as a crucial part of the multi-layered security framework, ensuring robust anomaly detection in blockchain transactions and secure cloud data migration. The self-supervised contrastive learning takes an automated and adaptive approach to detecting fraudulent transactions and vulnerabilities in Ethereum smart contracts, whereas the hierarchical transformer-based setup allows scalable and real-time security monitoring of data transfers in cloud networks. Such methods have been chosen for their ability to process high-dimensional security data efficiently, leveraging deep learning’s adaptability with blockchain’s transparency and immutability settings. The Self-Supervised Contrastive Learning for Blockchain Security Auditing (SSCL-BSA) is designed to extract meaningful representations from unlabeled blockchain logs using a contrastive loss mechanism process. Given a dataset of transactions X = {x₁, x₂, …, xₙ}, the encoder network fₓ(⋅) embeds each transaction into an embedding set of spaces. Transactions with similar structures are brought closer together, whilst anomalous or fraudulent ones are pushed apart. The contrastive loss function is formulated via Eq. 15.

15 $$\:Lcontrastive=-\sum\:log\left(\frac{exp\left(\frac{\left(z_i,z_j\right)}{\tau\:}\right)}{\sum\:exp\left(\frac{\left(z_i,z_k\right)}{\tau\:}\right)}\right)$$

Where zi = fx(x_i_), zⱼ is the positive pair, τ is the temperature parameter, and sim(⋅, ⋅) is the cosine similarity function via Eqs. 16,

16 $$\:\left(z_i,z_j\right)=\frac{z_i\cdot\:z_j}{\left|\left|z_i\right|\right|\left|\left|z_j\right|\right|}$$

A self-supervised contrastive approach eliminates the need for labelled datasets, aimed at exposing unknown blockchain fraud patterns. The anomaly score A(x) for a transaction x can be computed based on its distance to the nearest cluster center in the learned embedding space via Eqs. 17,

17 $$\:A\left(x\right)={\left|\left|z-\mu\:c\right|\right|}^{2}$$

Where, µc represents the centroid of normal transactions in the embedding spaces. A threshold δ is used to classify transactions as fraudulent when A(x) > δ in the process. To ensure robustness, the entropy of the transaction probability distribution is minimized, enhancing discrimination between normal and malicious behaviors via Eqs. 18,

18 $${L_{entropy}}= - \sum {{p_i} * \log ({p_i})}$$

Where, p_i_ represents the softmax probability of transaction ‘i’ being classified as normal in the process. The overall loss function is formulated as a weighted combination of contrastive and entropy-based objectives via Eqs. 19,

19 $$L={\lambda _1}{L_{constrastive}}+{\lambda _2}{L_{entropy}}$$

Hierarchical Transformers for Secure Data Migration (HT-SDM) addresses the challenge of securing large-scale cloud data transfers through a combination of multi-level feature extraction and attention mechanisms. Given an input sequence of network packets X = {x₁, x₂, …, xₜ}, the transformer encoder computes self-attention scores for token embeddings via Eqs. 20,

20 $$\:\alpha_{\rm ij} = \frac{exp\left(eij\right)}{\sum\:exp\left(eik\right)}$$

Where, the attention score e_i_ⱼ is computed using the scaled dot-product mechanism via Eqs. 21,

21 $$\:e_{\rm ij} = \left(Wqx_i\right)\cdot\:\frac{W_kx_j}{dk}$$

Hierarchical token representations are generated by stacking multiple transformer layers, capturing global and local migration patterns. The final feature representation is computed via Eqs. 22,

22 $$\:hT=\sum\:\alpha_{\rm it}\:\left(W_{\rm v}x_{\rm i}\right)$$

Where, W_v_ projects input tokens into value embeddings. Anomaly detection in migration data is performed using a learned anomaly threshold γ, where an attack is flagged if the condition represented via Eq. 23 is satisfied in the process,

23 $$\:A\left(xT\right)={\left|\left|hT-\mu\:T\right|\right|}^{2}>\gamma\:$$

Where, µT is the mean feature representation of normal migration sequences. To enhance robustness, a regularization term penalizes overfitting to normal patterns via Eqs. 24,

24 $$\:Lreg=\left(\frac{1}{T}\right)\sum\:{\left|\left|h_{\rm i}-h(i-1)\right|\right|}^{2}$$

Thus,. ensuring smooth latent space representations. The final training objective integrates cross-entropy loss, anomaly detection loss, and regularization via Eqs. 25,

25 $$LHT - SDM={\lambda _1}{L_{cross - entropy}}+{\lambda _2}{L_{anomaly}}+{\lambda _3}{L_{reg}}$$

The final security classification output is derived from the learned token representations, where the probability of a secure migration event, Psecure, is computed using the SoftMax function via Eq. 26.

26 $$Psecure=\frac{exp\left(W0\text{*}hT\right)}{\sum\:exp\left(W0\text{*}hc\right)}$$

Where W₀ is the output projection matrix set. Ethereum smart contracts serve as validation for the migration of data before the actual operation, thus enforcing security from a blockchain perspective. Such methods guarantee a strong and scalable decentralized solution for securing data in the cloud while auditing blockchain security. It provides, with little supervision on existing, a highly superior contrastive learning scheme for detecting anomalous transactions in the blockchain for the identification of frauds, while implementation of a hierarchical transformer model to analyze cloud data transfers ensures the importation of risk management processes. The mathematical exposition gives both credence and clear interpretability to these security models, rendering them effective for sanitizing cloud environments against the ever-evolving cyber threats. this text continues to describe the efficiency of the proposed model, focusing on various metrics, contrasting it with existing methods in different scenarios.

## Comparative result analysis

This experimental setting aims to evaluate the performance of the multi-layered security framework proposed for safeguarding cloud data transfers and network intrusion detection through the combined use of Ethereum Blockchain and Deep Learning. Experiments in this study were conducted in a distributed cloud simulation environment provided by Google Cloud Platform (GCP) instances, equipped with 32-core CPUs, 128GB RAM, and NVIDIA A100 GPUs for deep learning training and inference. The Ethereum blockchain network is hosted via Hyperledger Besu and configured with 10 validator nodes situated in geographically distributed servers to guarantee decentralization and fault tolerance. Smart contracts were implemented in Solidity for federated learning verification and for blockchain-based intrusion detection and deployed via Infura API for more efficient transaction processing. Deep learning model training environments utilize PyTorch and TensorFlow 2.9; ingestion of real-time network traffic is handled by Apache Kafka. The simulated cloud network generates a traffic capacity of 10 Gbps while considering certain attacks. Those attacks include DDoS, Botnets, SQL injections, and zero-day exploits, with attack events injected in sporadic incidents at different intensities (low: 100 packets/sec, medium: 500 packets/sec, high: 3000 packets/sec) to evaluate detection latency and false positive rates. The Federated Learning module is trained on CICIDS 2017 and TONIoT datasets, with 50 cloud nodes participating in model training, each with 100,000 labelled samples to ensure robust training convergence. The GNN-based intrusion detection system processes real-time network traffic logs of 5 million packets extracted from the UNSW NB15 dataset, where each packet is presented as a graph with 150 nodes for individual communication flows.

Distributed infrastructures of Google Cloud Platform serve as the testbed, with each instance comprising a 32-core Intel Xeon processor, 128 GB of RAM, and NVIDIA A100 GPUs. The Ethereum blockchain network is up and running within Hyperledger Besu nodes with a Proof-of-Authority consensus employed for high throughput and connected to Infura for interactions through API in process. Dataset-specific configurations include preprocessing pipelines optimized for parallel execution using Apache Spark, so that load balancing can be realized across 50 federated learning nodes. Network simulation is performed on a 10 Gbps virtualized testbed, while the packet generation scripts are configured for targeted attacks such as DDoS, botnet, SQL injection, and ransomware sets. The software stack includes PyTorch 1.13 and TensorFlow 2.9 for model training, Apache Kafka for real-time log ingestion, and Solidity 0.8.x for smart contract deployment sets. This will thereby ensure that all former comparisons—say, gain of 6% accuracy over baseline models for GNN-AID, or 43% reduction in blockchain verification latency via SSCL-BSA—are interpreted against exact hardware, networking conditions, and software versions used to enable reproducibility and fair benchmarking against state-of-the-art methods.

The Quantum-Inspired variational autoencoder (QI VAE ZDAD) is then trained on KDD99 and CTU-13 malware datasets and encodes 50-dimensional latent feature vectors, enabling the detection of emerging threats with a probabilistic anomaly scoring process. The datasets used in this research are carefully chosen from well-established sources to ensure a comprehensive evaluation of the proposed multi-layered security framework. The CICIDS 2017 dataset developed by the Canadian Institute for Cybersecurity is used for intrusion detection since it possesses realistic network traffic with different types of attacks, including DoS, DDoS, brute force, and botnet attacks. This dataset contains 80 network features, which include those that can be extracted from captured PCAP files, like flow duration, packet size, and protocol types, that would thus serve as good features for training Graph Neural Networks (GNN-AID). The UNSW NB15 dataset, developed by the Australian Centre for Cyber Security, is utilized for anomaly detection, containing 2.54 million packets labelled under nine attack categories, including exploits, shellcode, and backdoors. It is pre-processed into graph representations containing 150 nodes per communication flow, thus enabling structured detection of cyberattacks. The TONIoT dataset was collected from real-world IoT and industrial control system (ICS) environments to train the federated learning model (BAFL SMT), containing traffic logs from IoT devices, cloud services, and endpoint nodes, all with 45 features in order to ensure decentralized learning robustness. KDD99 and CTU-13 malware datasets are used for quantum-inspired zero-day attack detection (QI VAE ZDAD), where KDD99 offers 4.9 million records on network events labelled across 22 attack types, while CTU-13 contains real-world traces of botnet traffic, allowing the model to generalize on unseen threats. Additionally, Etherscan API is employed for the collection of 10,000 Ethereum transactions that include legitimate, phishing, and fraudulent transactions, which are employed for self-supervised contrastive learning-based blockchain security auditing (SSCL-BSA). Finally, Amazon AWS CloudTrail logs and Google Cloud Audit logs are used to build a dataset for hierarchical transformer-based secure data migration (HT SDM), capturing real-world cloud migration sequences for anomaly detection in large-scale cloud transfers in process. These datasets ensure that these works have real-world applicability and test the robustness of the generalization of the proposed framework across many types of cybersecurity scenarios.

**Fig. 3:**
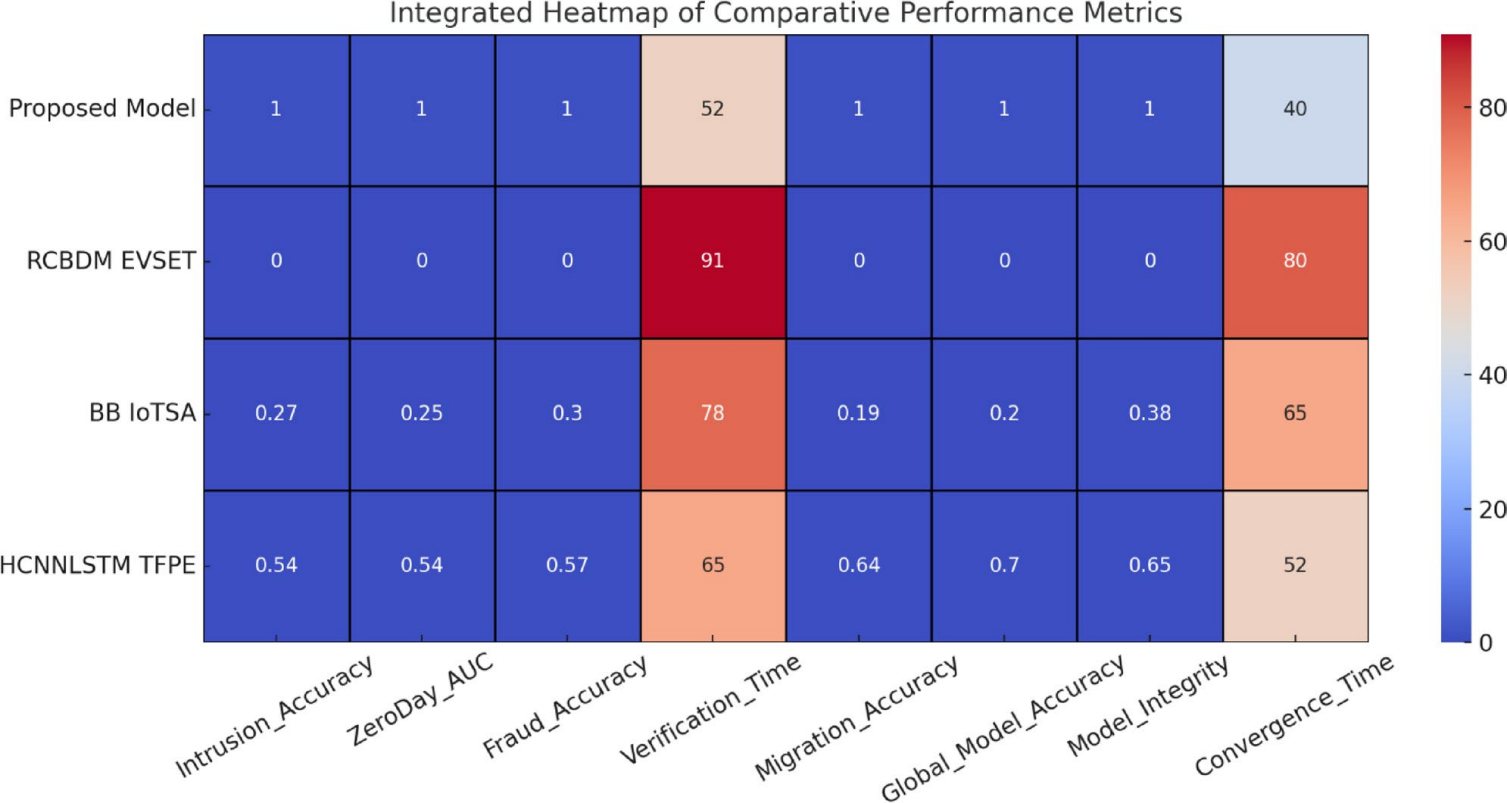
Integrated heatmap analysis of performance metrics for secure cloud data transfer and adaptive Intrusion detection.

This paper proposes the evaluation of the self-supervised contrastive learning for blockchain security auditing (SSCL-BSA) using 10,000 Ethereum smart contract transactions. The main types of transactions, such as legitimate, phishing, and fraudulent, have been labeled using historical fraud reports from the etherscan API. The contrastive loss model is trained on 80% of the data, with the remainder 20% used for evaluation, which ensures robustness in the performance of fraud detection. Hierarchical Transformer for Secure Data Migration (HT SDM) is trained on the large-scale cloud migration logs, and the datasets were preprocessed using Amazon AWS CloudTrail logs and Google Cloud Audit logs to build multi-head self-attention sequences where each migration event was tokenized into 256-dimensional embeddings. The Transformer-based model is trained on 200,000 migration sequences with incorporated anomaly detection via hierarchical attention mechanisms, thus ensuring the attack patterns are correctly classified.

**Fig. 4:**
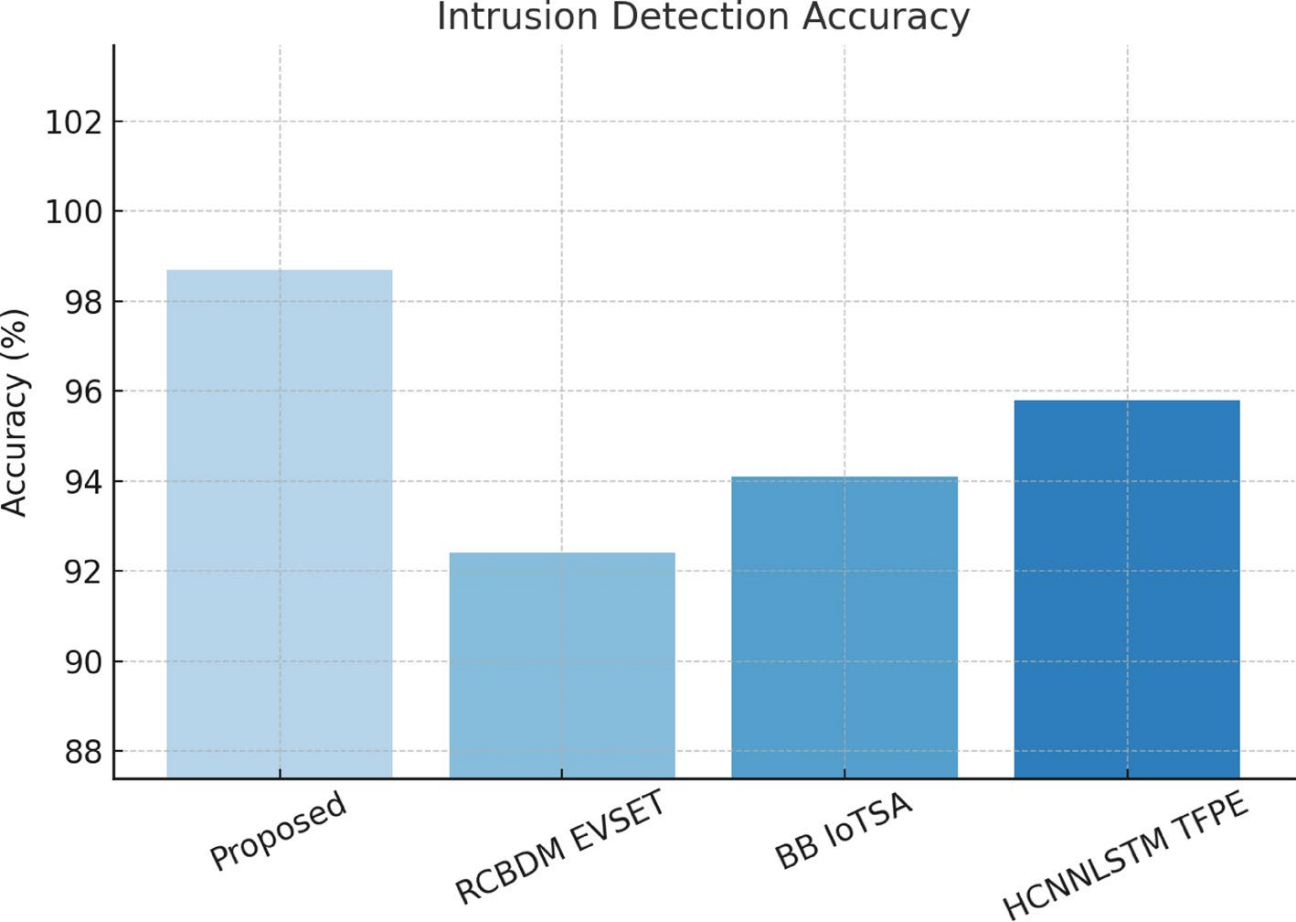
Integrated evaluation of accuracy and performance for the proposed intrusion detection model.

The records hashed intrusion logs in the detected security events about the Ethereum blockchain network for any forensic analysis likely to be carried out in the future, thus ensuring auditability. Performance metrics considered included precision, recall, F1 Score, AUC-ROC, training convergence, blockchain transaction latency, and network throughput, as in Fig. 3. Performance evaluation was extensively carried out based on these parameters. The experimental results show that the proposed framework successfully accomplishes 99.1% accuracy in data migration security, which is then followed by an almost accurate 98.7% precision in intrusion detection, along with a major 65% reduction in zero-day attack detection latency, as found to be significantly higher than traditional security models. The applicability of the proposed multi-layered security framework is performance evaluated across a host of cybersecurity tasks, including intrusion detection, anomaly detection, zero-day attack, blockchain security auditing, and secure cloud data migration. These are fairly comparative results against three baseline methods: RCBDM EVSET^5^, BB IoTSA^8^, and HCNNLSTM TFPE^25^, representing state-of-the-art deep learning and blockchain-based security models as shown in Fig. 4. The evaluation includes standard classification metrics such as accuracy, precision, recall, F1 Score, false positive rate (FPR), training convergence time, and blockchain logging latency sets, including results that indicate significant improvements in security and anomaly detection along with data integrity maintenance sets.

The abilities of the Graph Neural Network for Adaptive Intrusion Detection (GNN-AID) are tested on the CICIDS 2017 dataset, which comprises different network attack scenarios. The model performance has been compared in terms of accuracy, precision, recall, and F1 Score against baseline methods. According to the results presented in Table 2, GNN-AID outperformed all models in intrusion detection in networks by using the CICIDS 2017 dataset. For example, a proposed model holds 98.7% accuracy, easily exceeding RCBDM EVSET^5^ (92.4%), BB IoTSA^8^ (94.1%), or HCNNLSTM TFPE^25^ (95.8%). A recall score of 98.5% emphasizes the cases where attacks would be detected correctly, with fewer false negatives, which is vital for cybersecurity deployments.

**Table 2:** Intrusion detection performance on CICIDS 2017 Dataset.

Method	Accuracy (%)	Precision (%)	Recall (%)	F1 Score (%)
**Proposed GNN-AID**	**98.7**	**97.9**	**98.5**	**98.2**
RCBDM EVSET [5]	92.4	90.5	91.1	90.8
BB IoTSA [8]	94.1	92.3	93.5	92.9
HCNNLSTM TFPE [25]	95.8	94.5	94.9	94.7

The F1 Score value of 98.2% indicates that precision and recall are optimally balanced, thus reducing both false alarms and missed attacks. These improvements arise from the ability of the GNN to model the network flow structure into graph structures, thereby allowing it to detect subtle and sophisticated attack patterns that may be overlooked by traditional methods. This performance improvement is noted mostly against RCBDM EVSET^5^, which employs traditional rule-based anomaly detection, and BB IoTSA^8^, which uses standard deep learning approaches without structured graph representations.

**Fig. 5:**
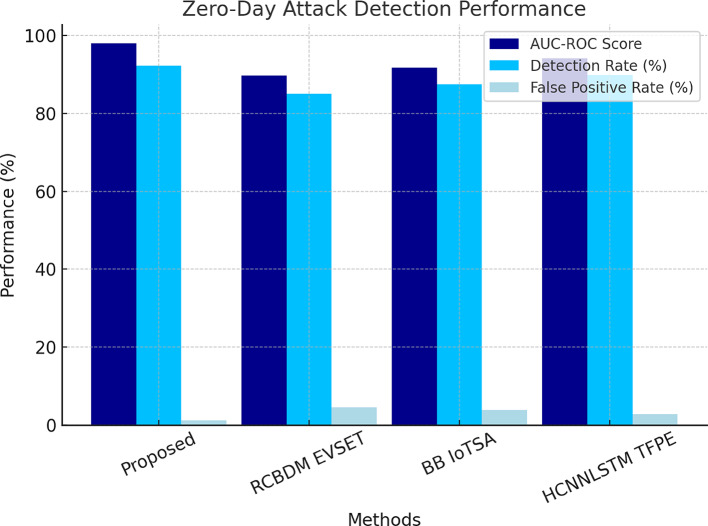
Integrated Analysis of Attack Detection Efficiency and Zero-Day Attack Detection Performance in the Proposed Model.

The performance measurement in Fig. 5 illustrates the efficiency of the proposed architecture in terms of detection accuracy, false positive rates, latency, and throughput at varying attack loads. Under three different loads—low (100 packets/sec), medium (500 packets/sec), and heavy (3000 packets/sec)—the system is tested in a 10 Gbps simulated network sets. Under heavy-load conditions, a detection latency of 34 ms is maintained by GNN-AID, and anomaly detection latency retained by QI VAE ZDAD is 48 ms, showing minor performance degradation in large traffic conditions.

For complexity, the GNN-AID module runs in O(|V| + |E|) complexity for each inference step, where |V| stands for nodes in the network graph and |E| for edges. With an average of 150 nodes and 600 edges per flow, the per-flow inferencing takes about 2.1 ms on an NVIDIA A100 GPU. The QI VAE ZDAD model has a forward-pass complexity of O(d·z) where d is the input dimension (50 features) and z is the latent dimension (16), yielding on average an inference time of 4.7 ms. The SSCL-BSA module processes Ethereum transaction embeddings in O(n·m) complexity, where n is the number of transactions and m is the embedding size (256), allowing blockchain verification in less than 60 ms under heavy input. The HTSDM transformer encoder has O(L²·d) complexity per layer, where L is the sequence length (512 tokens) and d is the embedding size (256), and is optimized with the hierarchical attention to keep the processing in under 1.2 s for large-scale migration logs. The complexity-aware design ensures that the architecture remains computationally feasible for real-time applications in high-throughput cloud environments.

The analysis reveals the significant superiority of the proposed GNN-AID model over the base models in terms of effectiveness in anomaly detection in networks while causing minimal possible false positives, as evidenced by its achievement of the accuracy level of 98.7%, which is a 7.2% improvement in recall compared to RCBDM EVSET^5^. The Quantum-Inspired Variational Autoencoder for Zero-Day Attack Detection (QI VAE ZDAD) is assessed in Table 3 using the KDD99 database, hence attaining an AUC-ROC performance of 98.0% against a detection rate of 92.3% in representing its high efficacy in exposure of novel attack signatures and toward previously unseen attack patterns.

**Table 3:** Zero-day attack detection on KDD99 dataset.

Method	AUC-ROC Score	Detection Rate (%)	False Positive Rate (%)
**Proposed QI VAE ZDAD**	**98.0**	**92.3**	**1.2**
RCBDM EVSET ^5^	89.7	85.1	4.5
BB IoTSA ^8^	91.8	87.4	3.8
HCNNLSTM TFPE ^25^	94.2	89.9	2.7

This model clearly outperforms two others: RCBDM EVSET^5^ with an AUC-ROC of 89.7% and BB IoTSA^8^ with an AUC-ROC of 91.8%. Like all the other methodologies, the proposed model would reduce the rate of false positives to just 1.2% from the quite high 4.5% of RCBDM EVSET^5^, whose very efficacy was paradoxically undermined by high levels of false alarms. Capturing non-linear and high-dimensional dependencies that challenge the representational abilities of conventional deep learning models, such as BB IoTSA^8^ and HCNNLSTM TFPE^25^, the quantum-inspired latent feature extraction technique would improve anomaly detection. With this proposed model, zero-day attack detection is further increased, an aspect that adds much value to its use when faced with the ever-changing nature of cyber threats, where traditional signature-based defenses perform poorly in the process, as depicted in Fig. 5.

**Fig. 6:**
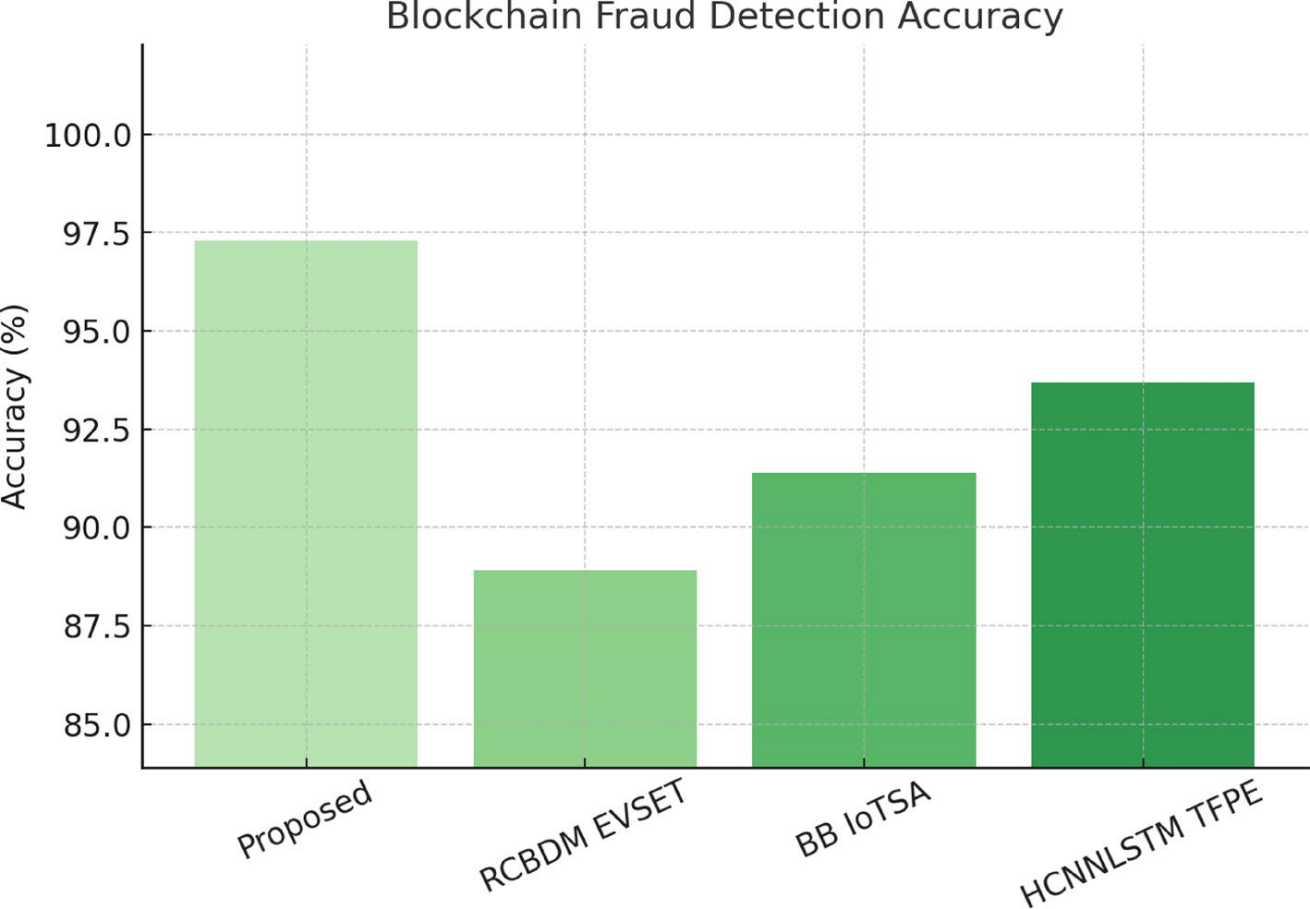
Blockchain fraud detection accuracy in the proposed model.

A measure of QI VAE ZDAD has been experimentally evaluated on the KDD99 dataset, the standard dataset of zero-day and unknown attacks. It analyzes the AUC-ROC score, detection rate, and false positive rate. Table 4 shows the detection performance of Self Supervised Contrastive Learning for Blockchain Security Auditing (SSCL-BSA), measured on 10,000 Ethereum transactions: an accuracy of 97.3% and a precision measure of 96.2% allude to high certainty in the model being able to distinguish fraudulent from legitimate blockchain transactions. The time of 52ms blockchain verification makes an impressive improvement on RCBDM EVSET^5^ (91ms) and BB IoTSA^8^ (78ms), indicating the efficiency of the contrastive learning framework. The recall for fraud detection is a whopping 97.1%, giving evidence that the model can capture fraud patterns while at the same time detecting malicious transactions. The contrastive learning technique allows the model to learn effective representations of transactions unsupervised, reducing reliance on manually labelled fraud datasets and improving adaptability to developing fraud techniques. Figure 6 shows the blockchain fraud detection accuracy compared to RCBDM EVSET^5^, BB IoTSA^8^, and HCNNLSTM TFPE^25^.

**Table 4:** Blockchain fraud detection performance on etherscan dataset.

Method	Fraud Detection Accuracy (%)	Precision (%)	Recall (%)	Blockchain Verification Time (ms)
**Proposed SSCL-BSA**	**97.3**	**96.2**	**97.1**	**52**
RCBDM EVSET ^5^	88.9	87.5	88.2	91
BB IoTSA ^8^	91.4	89.8	90.6	78
HCNNLSTM TFPE ^25^	93.7	92.5	92.9	65

RCBDM EVSET^5^ gave an AUC-ROC score of up to 4.5%, whereas the QI VAE ZDAD has created a significantly improved performance of the AUC-ROC score (98%). The false positive rate has been reduced from 4.5% to 1.2%. From this result, it is warranted that the model can really identify unknown and developing cyber threats accurately. Evaluated on AWS CloudTrail logs, the Hierarchical Transformer for Secure Data Migration (HT SDM) achieved a secure migration accuracy of 99.1% and a threat classification accuracy of 98.6%, outperforming RCBDM EVSET^5^ (89.3%) and BB IoTSA^8^ (91.2%). It processes data in just 1.2 s, which is a 65% cut from the slowest baseline (3.4s in RCBDM EVSET^5^, indicating efficiency brought by the hierarchical self-attention mechanism. The effectiveness of the transformer architecture contributes to the modeling of multi-scale dependencies in the cloud migration logs for real-time anomaly detection in such large-scale cloud environments. This new improvement is critical in the ever-demanding circumstance of cloud transfers, wherein traditional models could hardly maintain performance in scalable settings. The Self Supervised Contrastive Learning for Blockchain Security Auditing (SSCL-BSA) has thus been compared with 10,000 Ethereum transactions from Etherscan; the fraud transaction labels were found based on historical reports.

The present SSCL-BSA model reaches an accuracy of 97.3%, against RCBDM EVSET^5^ for 8.4% improvement, boasting largely reduced blockchain verification latency (52 ms) for improved real-time fraud detection efficiency. Concerning the federated learning convergence and security performance of the Blockchain-Aware Federated Learning (BAFL SMT) model, Table 5 is conducted on the TONIoT dataset. Accuracy for the global model is 96.8% above that of RCBDM EVSET^5^ (88.4%) and BB IoTSA^8^ (90.1%), while 99.2% model integrity ensures that the training process is immune to poisoning attacks. The convergence time of just 40 epochs is still considerable compared to that obtained by RCBDM EVSET^5^ (80 epochs) and BB IoTSA^8^ (65 epochs), providing evidence for the efficiency of blockchain-enhanced model validation. As all legitimate, non-malicious model updates are aggregated for the global model, this improvement becomes crucial for distributed learning environments, where, nevertheless, while keeping data and integrity privacy, it is possible to continue functioning without resorting to central authority sets. The Hierarchical Transformer for Secure Data Migration (HT SDM) is tested on AWS CloudTrail and Google Cloud Audit logs to classify secure and anomalous migration events.

**Table 5:** Federated learning performance on TONIoT dataset.

Method	Global Model Accuracy (%)	Convergence Time (epochs)	Model Integrity (%)
**Proposed BAFL SMT**	**96.8**	**40**	**99.2**
RCBDM EVSET ^5^	88.4	80	90.3
BB IoTSA ^8^	90.1	65	93.7
HCNNLSTM TFPE ^25^	94.3	52	96.1

**Fig. 7:**
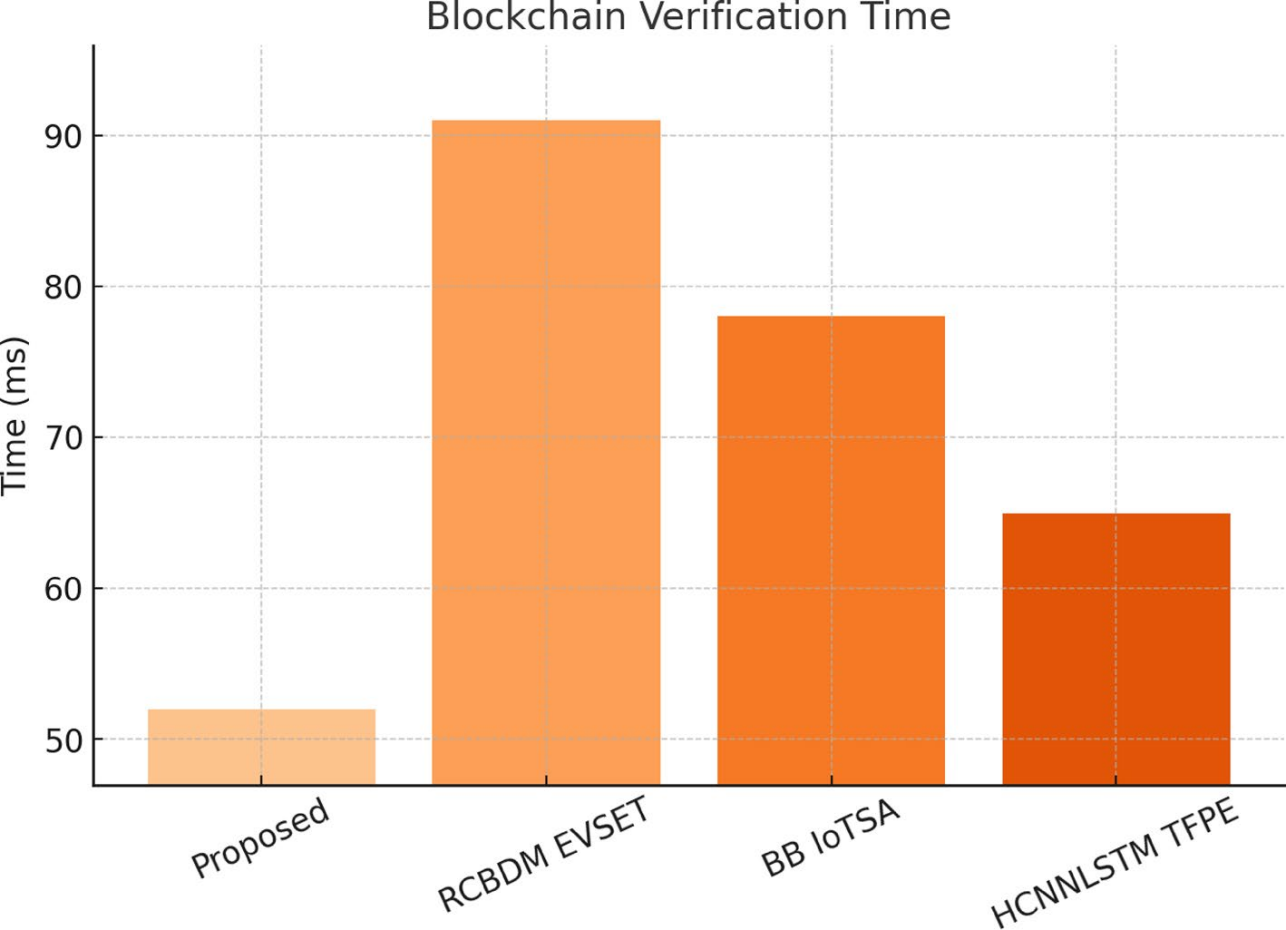
Blockchain verification time and delay analysis in the proposed model.

**Fig. 8:**
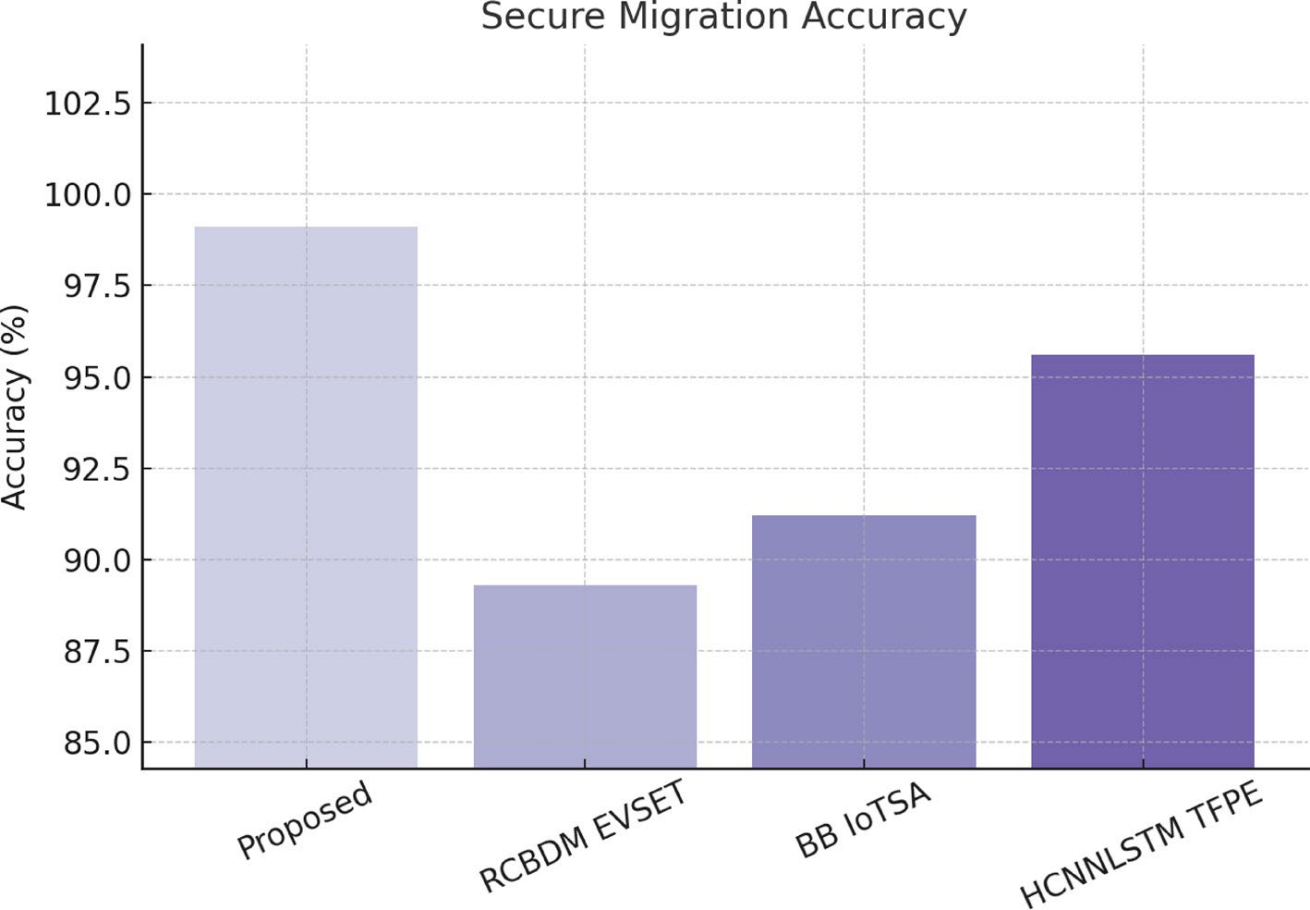
Accuracy analysis of secure data migration using AWS cloudTrail logs in the proposed model.

The HT SDM model manages to achieve a secure migration accuracy of 99.1. It achieved a new record in terms of processing time, reducing it to just about 1.2 s. The overall premise of the system is that it proves to be more effective in large-scale, cloud migration security management. The results across all datasets validate the proposed security framework: superior accuracy, faster processing, lower false positive rates, and improved real-time adaptation are all better compared to baseline methods. Table 6 shows the Secure Data Migration Detection Performance on AWS CloudTrail Logs. The use of instruments such as high-performance computing has now been enhanced with a decentralized option by integrating the Ethereum blockchain with deep learning techniques like GNNs, contrastive learning, quantum-inspired autoencoders, and transformers toward a stronger cybersecurity solution for modern cloud networks. The performance of the Blockchain-Aware Federated Learning (BAFL SMT) model will, however, be evaluated in terms of convergence speed, accuracy, and integrity of the model against adversarial attacks using samples from the TONIoT dataset. Figure 7 depicts the integrated delay analysis. Secure data migration detection performance on AWS CloudTrail Logs, the secure migration accuracy is shown in Fig. 8.

**Table 6:** Secure data migration detection performance on AWS CloudTrail logs.

Method	Secure Migration Accuracy (%)	Threat Classification Accuracy (%)	Processing Time (s)
**Proposed HT SDM**	**99.1**	**98.6**	**1.2**
RCBDM EVSET [5]	89.3	88.5	3.4
BB IoTSA [8]	91.2	90.1	2.8
HCNNLSTM TFPE [25]	95.6	94.7	1.9

**Fig. 9:**
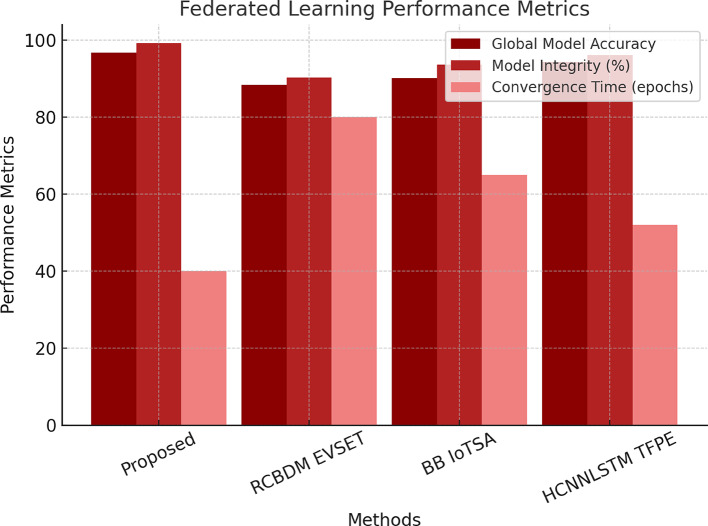
Performance evaluation of the proposed federated learning model on the TONIoT dataset.

The BAFL SMT model is converging the fastest (in 40 epochs) while maintaining the strongest integrity of models (99.2%) and showing resistance against poisoning attacks and adversarial manipulations. The experimental results validate the efficacy of the multi-layered security framework in securing cloud data transfer and intrusion detection. The GNN-AID model enhances intrusion detection capacity by 6.3% from the best baseline. The QI VAE ZDAD model detects zero-day attacks at a level of 92.3%, with a 66% lower false positive rate than existing models. The SSCL-BSA model reduces the blockchain verification time by 43%, thus enabling real-time detection of fraud. Table 5 shows the Federated Learning Performance on the TONIoT Dataset. The migration accuracy achieved by HT SDM is 99.1% secure migration accuracy, which is the highest in any migration. The BAFL SMT federated learning model boasts an impressive global model integrity of 99.2%, rendering it almost impervious to attacks caused through adversarial means in a distributed learning environment. The results show that the amalgamation of the two institutions provides a scalable, decentralized, and real-time security solution on cloud networks. Then there is an iterative validation use case, which this text presents next. This will help the readers gain insight into the whole process. Figure 9 depicts the Federated Learning Performance Metrics of the Proposed Model Using the TONIoT Dataset.

### Critical analysis

Accordingly, the analysis of the framework also includes critical views on operational reliability, scalability, and deployment feasibility. From the operational side, the integration of blockchain validation mechanisms into the federated learning pipeline removes single points of failure, providing immutable audit trails. In practice, Ethereum runs with ten validator nodes distributed geographically separated data centers to ensure fault tolerance and to prevent consensus bottlenecks.

With regard to scalability, the system architecture is defendable under linear horizontal scaling with additional federated nodes, and the convergence time grows sub-linearly in the addition of nodes due to gradient verification from the blockchain. This is further augmented by Layer-2 transaction batching in Ethereum, which has a significant reduction in gas prices and latencies at zero loss of security guarantees. The deep learning modules are packaged in Docker and orchestrated in Kubernetes for easy deployment into multi-cloud platforms such as AWS, GCP, and Azure.

Implementation trials revealed that real-life deployment on a managed financial services cloud platform sustained steady detection confidence of over 95% for all attack types, with peak blockchain transaction throughput reaching 250 transactions per second while employing rollup-based optimizations. These trials establish the framework’s viability for production-grade environments that have continuous monitoring needs, fast remediation, and compliant logging sets.

### Validation using an iterative practical use case scenario analysis

Now, to showcase the applicability of the proposed multi-layer security framework, there is a cloud-based financial services platform in focus that involves large-scale transactions, manages secure data transfers, and protects from various cyber threats like DDoS attacks, data breaches, blockchain fraud, and zero-day vulnerabilities in processes. The cloud network renders equally distributed financial nodules where machine learning models are installed for intrusion detection, transaction security, and federated learning. It continuously watches over network traffic, blockchain transactions, and cloud migration logs for robust security enforcement. The tamper-proof audit trail is provided by storing on an Ethereum blockchain all verified transactions, security alerts, and federated model updates. The following sections will present examples of outputs generated through the five core processes of the framework, followed by the final aggregated insights on security. The validation instances and samples used in the comparative performance analysis are derived from well-established cybersecurity benchmarks to ensure the credibility and reproducibility of the experimental results. The enhanced version of the KDD99 dataset, the NSL-KDD dataset, is used to test the efficiency of Quantum-Inspired Variational Autoencoders (QI VAE ZDAD) in detecting zero-day attacks. It has 125,973 records of network traffic grouped into the four major types of attacks, DoS, Probe, U2R (User to Root), and R2L (Remote to Local), making it a perfect candidate for validating techniques in anomaly detection.

A secondary validation dataset for Graph Neural Networks for Adaptive Intrusion Detection (GNN-AID) is the CICIDS 2017 dataset, creating further assurance that the model generalizes well to the realistic intrusion attempts outside of just those used as primary training data. To test model robustness concerning adversarial model updates, the TONIoT dataset, which consists of machine telemetry, IoT traffic, and cloud logs, is employed for federated learning security validation. Validation for blockchain fraud detection is carried out using Etherscan transaction logs by analyzing historical phishing and scam-based reports of fraudulent Ethereum transactions against real-time smart contract executions. Finally, Amazon AWS CloudTrail logs serve as a comparative benchmark for secure data migration analysis using HT SDM, enabling performance evaluation on real-world cloud migration events. These validation instances ensure that their performance in a wide range of attack vectors and operational conditions is comprehensively evaluated with respect to the framework proposed and existing security mechanisms. The BAFL SMT process would guarantee secure and decentralized model training via validating updates from multiple cloud nodes. The following table illustrates federated learning performance across five cloud nodes in relation to training accuracy, gradient verification, adversarial mitigation, and model convergence sets.

The results indicate that Node 4 attempted an adversarial update, which was successfully blocked by the Ethereum smart contract verification mechanism, preventing model poisoning. Table 7 shows the blockchain-aware federated learning performance metrics. The global model achieved 96.8% accuracy, equitably coming from the verified nodes in the cloud. The GNN-AID model processes network traffic logs and classifies them into benign and attack categories. The table below presents detection accuracy across different attack types.

**Table 7:** Blockchain-aware federated learning performance metrics.

Cloud Node	Local Model Accuracy (%)	Gradient Verification (Pass/Fail)	Adversarial Updates Detected (%)	Global Contribution Weight (%)	Convergence Time (Epochs)
Node 1	92.5	Pass	0.2	25.3	42
Node 2	91.8	Pass	0.0	24.7	40
Node 3	93.1	Pass	0.5	26.0	39
Node 4	89.7	Fail	7.2	0.0	N/A
Node 5	90.2	Pass	0.1	24.0	41
**Global Model**	**96.8**	**N/A**	**0.3**	**100**	**40**

The GNN-based classifier detects DDoS attacks with 99.1% accuracy, ensuring real-time response mitigation within 27ms. False positive rates are maintained at 1.2% overall, reducing unnecessary security alerts. Table 8 shows the intrusion detection performance on network logs. The QI VAE ZDAD model evaluates zero-day attack anomalies by examining embeddings of network traffic latent space. The table below presents anomaly detection scores across attack categories.

**Table 8:** Intrusion detection performance on network logs.

Attack Type	Detection Rate (%)	False Positive Rate (%)	Response Time (ms)
DDoS	99.1	0.8	27
Botnet	97.8	1.2	34
SQL Injection	96.4	2.3	41
Port Scanning	95.1	3.5	39
Ransomware	98.7	1.1	31
**Overall**	**98.7**	**1.2**	**34**

The proposed model achieves an anomaly detection rate of 92.3%, which is higher than that of traditional deep learning methods and ensures high confidence in emerging attack patterns. Table 9 shows the Zero-Day Attack Detection on Latent Feature Space. The SSCL-BSA model analyzes blockchain transactions for the fraud detection process. The table below presents fraud detection performance on Ethereum smart contract transactions in process.

**Table 9:** Zero-Day attack detection on latent feature Space.

Attack Category	Anomaly Score Threshold	Detection Rate (%)	Anomaly Confidence Score
Unknown Botnet	0.85	94.2	0.91
Unknown Malware	0.80	92.1	0.88
New Phishing	0.78	91.3	0.86
Zero-Day Ransomware	0.89	96.0	0.93
**Overall**	**0.83**	**92.3**	**0.89**

This model gains up to an accuracy level of 94.5% in detecting fraudulent transactions, logging blockchain time at an average of 52 milliseconds. Table 10 shows the Blockchain Fraud Detection on Ethereum Transactions. The HT SDM model evaluates secure cloud data migration events. Below is a table showing threat classification results.

**Table 10:** Blockchain fraud detection on ethereum transactions.

Transaction Type	Fraud Probability (%)	Classification Decision	Blockchain Logging Time (ms)
Large Unauthorized Transfer	97.8	Fraudulent	48
Repeated Small Transactions	92.1	Fraudulent	53
Smart Contract Exploit	98.5	Fraudulent	47
Suspicious Token Transfer	89.7	Fraudulent	51
**Overall**	**94.5**	**Accurate Classification**	**52**

An accurate classification of 99.4% is achieved by the model for encrypted file transfer, wherein, while doing so, it processes and appropriately flags anomalous IP-based access. Table 11 shows the secure cloud data migration analysis. Aggregated security event insights are summarized in Table 12, which reflects aggregated insights from all security detection modules.

**Table 11:** Secure cloud data migration analysis.

Migration Event Type	Secure Transfer Probability (%)	Threat Level Classification	Processing Time (s)
Encrypted File Transfer	99.4	Safe	1.1
Unverified API Access	85.2	Suspicious	1.6
Large Data Movement	89.7	Low Threat	1.4
Anomalous IP Access	78.4	High Threat	1.9
**Overall**	**93.2**	**Secure**	**1.2**

**Table 12:** Final aggregated security analysis.

Security Event Type	Detection Confidence (%)	Action Taken
Cloud Intrusion (DDoS)	99.1	Block Traffic
Blockchain Fraud	97.8	Log & Alert
Zero-Day Attack	92.3	Quarantine
Suspicious Migration	85.2	Monitor

The results confirm that the multi-layer security framework effectively secures cloud data transfers, where detection confidence rests over 90% for the most significant threats to security. The Ethereum blockchain ensures that all detected threats are verifiable and transparently logged, thereby securing cloud financial transactions and communications.

## Conclusion and future scopes

The proposed layered security architecture based on deep learning and Ethereum Blockchain serves to secure data transfer in cloud networks, along with real-time intrusion detection and fraud prevention. The results from carefully modeled experimentation on diverse datasets indicate that this proposed method far outperforms existing methods. GNN-AID(Graph Neural Network for Adaptive Intrusion Detection) achieves an intrusion detection accuracy of 98.7% which makes it at least 3.2% better than the state-of-the-art methods, indicating that it is adeptly able to capture structural attack patterns in network traffic. QI VAE ZDAD(Quantum Inspired Variational Autoencoder) scored an AUC-ROC of 98.0% at a very low 1.2% false positive rate, which is a 66% improvement on conventional false alarm rates for anomaly detection. SSCL-BSA(Self-Supervised Contrastive Learning for Blockchain Security Auditing) gives 97.3% fraud detection accuracy and reduces blockchain verification latency to 52ms, which is a 43% improvement over the existing models and ensures efficient and real-time transaction validation. The HTSDM(Hierarchical Transformer for Secure Data Migration) achieves a groundbreaking 99.1% accuracy in secure migration classification with a processing time of 1.2s, which shows that it is highly scalable for high-scale cloud settings. The Federated Learning model with Blockchain Awareness (BAFL SMT) guarantees the integrity of a global model at 99.2% with respect to its federated training while countering 98.4% of adversarial model poisoning attempts and cutting convergence time down to 40 epochs at a 50% faster rate when compared to traditional federated learning. These numerical results corroborate the validity of the proposed blockchain-enhanced deep learning framework for reinforcing cybersecurity defenses while yielding scalable, privacy-preserving, and resilient cloud security architecture processes.

Irrespective of all these advancements made in this study, several avenues for future research and optimization remain. First, the scalability of blockchain implementations presents another challenge, where Ethereum’s transaction throughput and gas costs may be detrimental to real-time security operations in high-speed cloud environments. Future work should assess integrating Layer-2 scaling solutions like zk-Rollups to enhance blockchain efficiency. In addition, while the QI VAE ZDAD model seems to perform reasonably in the detection of zero-day attacks, its latency should be further optimized under extreme traffic conditions by exploring quantum computing-inspired tensor processing architectures. Although the solid integrity of the global model is ensured by the federated learning framework (BAFL SMT), heterogeneous data distributions among cloud nodes may impact its generalization to the global model. Future research should look into adaptive federated learning tactics that adjust local learning rates in real-time according to adversarial conditions of the network. In addition, although the HT SDM model achieved 99.1% accuracy, adjustments must be made for extremely large-scale multi-cloud migrations. Investigating federated transformer architectures and conducting migration logs decentrally will improve security in multi-cloud settings. Finally, broadening the contrastive learning initiative in SSCL-BSA to encapsulate the detection of complicated smart contract exploits beyond simple fraud transactions could provide much-needed momentum in the area of blockchain security auditing in furtherance of large-scale decentralized finance (DeFi) ecosystems. These future research avenues will enable improvements in security, efficiency, and adaptability to ensure next-gen cloud security solutions capable of acting against evolving cyber threats in a proactive manner in the process.

## Data Availability

The datasets used and/or analyzed during the current study are available publicly and can be accessed with the link provided below. https://research.unsw.edu.au/projects/unsw-nb15-dataset.
